# Bi-directional electrical recording and stimulation of the intact retina with a screen-printed soft probe: a feasibility study

**DOI:** 10.3389/fnins.2023.1288069

**Published:** 2024-01-08

**Authors:** Ieva Vėbraitė, Chen Bar-Haim, Moshe David-Pur, Yael Hanein

**Affiliations:** ^1^School of Electrical Engineering, Tel Aviv University, Tel Aviv, Israel; ^2^Sagol School of Neuroscience, Tel Aviv University, Tel Aviv, Israel

**Keywords:** bi-directional electrophysiology, electrical stimulation, intact retina, soft neural interface, neurostimulation, neural prosthesis

## Abstract

**Introduction:**

Electrophysiological investigations of intact neural circuits are challenged by the gentle and complex nature of neural tissues. Bi-directional electrophysiological interfacing with the retina, in its intact form, is particularly demanding and currently there is no feasible approach to achieve such investigations. Here we present a feasibility study of a novel soft multi-electrode array suitable for bi-directional electrophysiological study of the intact retina.

**Methods:**

Screen-printed soft electrode arrays were developed and tested. The soft probes were designed to accommodate the curvature of the retina in the eye and offer an opportunity to study the retina in its intact form.

**Results:**

For the first time, we show both electrical recording and stimulation capabilities from the intact retina. In particular, we demonstrate the ability to characterize retina responses to electrical stimulation and reveal stable, direct, and indirect responses compared with *ex-vivo* conditions.

**Discussion:**

These results demonstrate the unique performances of the new probe while also suggesting that intact retinas retain better stability and robustness than *ex-vivo* retinas making them more suitable for characterizing retina responses to electrical stimulation.

## 1 Introduction

Electrophysiological investigations of the retina tap into the electrical activity of the cells in the retina, particularly the neurons that transmit the signals they receive from the photoreceptors to the brain. This electrical activity is influenced by various signaling pathways and reflects on the retina's developmental stage, circuitry, and viability. The knowledge gained from electrophysiological interrogation of the retina is important for understanding underlying mechanisms of retinal function and connectivity. Non-invasive studies of the electrophysiology of the retina involve techniques such as electroretinography (ERG), which measures the electrical response of the retina to light stimulation, and pattern electroretinography (PERG), which assesses the response of the retina to specific visual patterns (Creel, 2019; Cornish et al., 2021). These tests can be used to evaluate the function of the retina and to diagnose various retinal disorders, such as macular degeneration and retinitis pigmentosa, among others (Moschos and Nitoda, 2018; Menghini et al., 2020). ERG is widely used on animal models in basic and applied research (Vinberg and Kefalov, 2015; Pasmanter and Petersen-Jones, 2020). Despite their many benefits, non-invasive approaches suffer from inherently low resolution.

High-resolution insight into retina electrophysiology can be gained with *ex-vivo* retinas (retinas that have been surgically removed from an animal and maintained *in vitro*) as a model system for vision research. There are several benefits regarding *ex-vivo* retinas as a model system: *Ex-vivo* retinas retain their *in vivo* organization and structure, including the layers of cells and their connections, energy metabolism, gradients of electrolytes, and amino acids (Ames and Nesbett, 1981). This relative integrity allows the study of the behavior of retinal cells in a physiologically relevant environment. *Ex-vivo* retinas can be used to study the structure, function of the retina, and its response to different stimuli, such as light, drugs, and electrical stimulation (Field et al., 2010; Madugula et al., 2022; Shah et al., 2022). *Ex-vivo* retinas can be used to study retinal diseases and their effects on retinal function and structure, thus providing important information needed in the development of new treatments for retinal degenerative diseases (Chang et al., 2019; Chenais et al., 2019; Tong et al., 2020). However, *ex-vivo* retinas have limitations as a model system. For example, they may not sustain their full functionality over time, due to environmental changes and the effects of tissue dissection. High-resolution strategies to study the electrophysiology of the intact retina are therefore appealing to explore the differences between the intact and *ex-vivo* retinas (Vėbraitė and Hanein, 2022).

Although studies on soft neural probes exist, none to the best of our knowledge, documented bi-directional high-resolution electrophysiological investigation of the intact retina. Several studies described flexible electrode arrays that are potentially suitable for bidirectional retina stimulation and recording, presenting preliminary testing with *ex-vivo* retinal model (not *in-vivo*) or chronic biostability testing (Rodger et al., 2008; Montes et al., 2019). Others discussed high-density ultra-flexible electrode arrays for chronic cortical recordings (Zhao et al., 2023). Nevertheless, proven high-resolution tools to study the electrophysiology of the intact retina are presently scarce and only a few electrophysiological investigations of the intact retina were described. We refer the interested reader to a mini review on this specific topic (Vėbraitė and Hanein, 2022). In fact, when reviewing the entire expansive literature on soft neural technologies, it is apparent that these technologies manifest either high-quality stimulation or recording (Vėbraitė and Hanein, 2021), not both.

Multi-electrode arrays designed for the bi-directional study of the intact retina demand soft probes with small electrode dimensions that can record and stimulate the retina at high fidelity. The probe properties should include bio-compatibility, durability, and sterilization compatibility. Such retinal probes should not cause damage to the gentle tissue, and for some applications, optical clarity is important (Vėbraitė and Hanein, 2021; Zheng et al., 2021; Oldroyd and Malliaras, 2022). Finally, electrode material has to exhibit stability during stimulation (Schiavone et al., 2020).

In this study, we introduce an innovative method to fulfill these requisites. The focus of this paper is the development of a screen-printed soft multi-electrode array for bi-directional neural interfacing. To showcase the potential of this technology as a neural interface, we illustrate its application in studying the intact retina.

Flexible high-resolution neural probes are conventionally realized using photolithography and thin film technology, which are ideal for high-resolution device fabrication (Herwik et al., 2009; Boehler et al., 2017). However, under physiological conditions, these probes tend to suffer from poor stability, delamination (Boehler et al., 2017), and relatively high rigidity, imposed by substrate material (e.g., polyimide) and compromised electrochemical properties. Moreover, soft neuronal electrodes tend to manifest poor recording performances (Vėbraitė and Hanein, 2021). The approach we describe here builds on stacked screen-printed carbon on soft polyurethane (PU) films. Polyurethane, as a substrate and passivation material, offers low Young's modulus (in the 7–30 MPa range), low water diffusion coefficient (3.18 × 10^−10^*m*^2^*s*^−1^), breathability, compatibility with sterilization methods, and adherence to ISO 10993-1 toxicity standards (Rezaei et al., 2010; Vėbraitė and Hanein, 2021; Materials and Technologies, 2022). A screen-printed carbon-based approach resolves several limitations of photolithography-based thin-film probes: Carbon electrodes are characterized by high stability, they are non-Faradaic (within a range allowing safe stimulation), while also having favorable electrochemical properties to enable high-resolution recording.

Our previous work in Vėbraitė and Hanein (2022) demonstrated the feasibility of capturing high-quality spontaneous and light-induced signals from *ex-vivo* retinal tissue using soft carbon probes, an improvement over traditional titanium nitride (TiN) multi-electrode arrays as shown in Vėbraitė et al. (2021). Expanding on this technological foundation, we have taken a step forward by describing a soft multi-electrode interface with 36 channels, enabling both the recording and stimulation of the intact retina in a two-way manner. The fabrication process relies on the layering of two layers of screen-printed carbon, resulting in stable electrode arrays. To enhance the electrochemical attributes of the electrodes, we incorporated plasma polymerization of EDOT.

The study also encompasses electrochemical measurements, which were conducted to characterize the properties of these electrodes. Furthermore, a feasibility study to showcase the practical application of these devices for the simultaneous recording and stimulation of the intact chick retina is presented. This approach reveals responses within the intact retina, most notably an enhancement in stability over extended periods of stimulation and recording. Finally, our study underscores distinctions between the conditions of an intact retina and an *ex-vivo* retina, shedding light on the implications of each setting.

## 2 Materials and methods

### 2.1 Materials

Materials were purchased from Sigma-Aldrich unless specified otherwise. Phosphate buffered saline (PBS: 2.7 mM KCl, 137 mM NaCl, 10 mM phosphate buffer, pH 7.4). Artificial cerebrospinal fluid (aCSF: 5 × 10^−3^ M KCl, 25 × 10^−3^ M NaHCO_3_, 9 × 10^−3^ M glucose, 1.2 × 10^−3^ M MgSO_4_, 1.2 × 10^−3^ M HEPES, 0.5 × 10^−3^ M glutamine, 2.5 × 10^−3^ M CaCl_2_, pH 7.2). Materials for electrodes include polyurethane film (9,832 F, 3M Medical Specialties), single-coated polyurethane medical tape (9,832 W, 3M Medical Specialties), carbon screen print ink (C200, Kayaku Advanced Materials), 3,4-ethylenedioxythiophene (EDOT).

### 2.2 Electrode fabrication

Electrodes were fabricated using screen printing of carbon on polyurethane (PU) films as described previously (Vėbraitė et al., 2021). Screen printing is inherently a low-resolution technology, thus pre-patterned mesh stencil (Sefar Inc.) properties were customized to improve printing resolution achieving line widths as low as 40 μm. Furthermore, to achieve high electrode density in a small device area (1–4 mm^2^), layer stacking was employed. The device consists of three layers: Layer 1 (L1) consisting of PU film (20 μm thick) with screen-printed carbon traces (60 μm in diameter), Layer 2 (L2) consisting of single-sided adhesive PU film (50 μm thick) with screen printed carbon traces (60 μm in diameter). L2 also had a laser-cut circular hole (990 μm in diameter) to expose electrodes in L1, and Layer 3 as a passivation layer consisting of single-sided adhesive PU film (50 μm thick) with laser-cut circular holes (1,050 μm to expose electrodes in L1 and 50 μm holes to expose electrodes in L2). Each layer was prepared separately then the three layers were aligned and pressed together under a microscope (Leica M420). In total 36 traces were integrated at 150 μm pitch: 32 recording micro-electrodes (50 μm in diameter), two additional stimulating electrodes (50 μm in diameter), and two big reference electrodes.

We refer to these electrodes as “SoftC probe” to reflect the soft polyurethane substrate, and the conductive material used (carbon, hence “C”). The total SoftC probe thickness was 160 μm. A custom-made flexible printed circuit board (PCB) was used to connect the soft probes with a connector (Omnetics). The binding between the PCB and the soft array was achieved with a z-axis adhesive. Detailed schematics of SoftC probe fabrication can be found in Supplementary Figure 1. In total 10 complete devices were fabricated and five were characterized (impedance values and surface area, see details below). Additional test structures were fabricated for surface and electrochemical analysis.

#### 2.2.1 Screen-printing

Screen-printing of carbon electrodes was achieved using a pre-patterned mesh stencil. Mesh stencil properties were customized to improve printing resolution. A mesh (360 wires/inch and 0.0006-inch wire diameter) with 5 μm emulsion over mesh (EOM) stretched at a 22-degree angle (SEFAR) was used. The screen was tramp mounted for extra reinforcement and a 70 Durometer squeegee was used to screen print the ink. Printing was accomplished by a manual application of conductive carbon ink on a paper-supported PU film. The ink particle size was < 10 μm, and the mesh opening was 56 μm, allowing the ink to pass without clogging the mesh. The printing step was followed by curing at 130°C for 10 min.

#### 2.2.2 Laser cutting

A single-sided adhesive PU layer was used to first passivate Layer 1 and Layer 2 electrodes. A laser cutter (ELAS Ltd.) was used to define circular holes: 990 and 1,050 μm in diameter in the center for L1 and L2, respectively, and 50 μm in diameter holes to expose electrodes in L2. A two-step process was used to prevent overheating of the adhesive layer: first with a laser intensity of 400 mW to remove the paper support layer, followed by an intensity of 800 mW to remove the remaining two layers (PU and plastic cover).

#### 2.2.3 Plasma polymerized EDOT coating

To complete the electrode fabrication, a plasma polymerized 3,4-ethylenedioxythiophene (ppEDOT) coating was applied. The process was performed using an RF plasma system (Pico-RF-PC, Diener electronics), operating at a frequency of 13.56 MHz and a monomer vapor pressure of 0.1 mbar. A plasma power of 90 W for 15 min was used.

### 2.3 Surface characterization

Electrode's approximate diameter and area were estimated and averaged over five SoftC probes from optical microscopy images with Image J software. To investigate the surface profile of the films, environmental scanning electron microscopy (ESEM) and confocal scanning laser microscopy (CSLM, Lext OLS3000) were used. The surface roughness was investigated using CSLM, root-mean-square (RMS) roughness (Rq) and height values were calculated. The surface wettability was characterized by the water contact angle using the sessile drop method. The contact angles of 2 μL deionized water droplets were measured at room temperature (RT) using a contact angle meter (Rame-Hart model 400). Contact angle values were calculated using DROPimage Pro software. Contact angle values were derived from the mean of six droplets deposited randomly at different locations.

### 2.4 Electrochemical characterization

The electrochemical properties of test electrode arrays (1, 2, 3, and 4 mm in diameter) were characterized using cyclic voltammetry (CV) in PBS at scan rates 15, 25, 35, and 45 mV/s within the −0.5 and 0.5 V range which was estimated to be within the water window limits. The CV characterization was done using a three-electrode cell configuration with an Ag/AgCl reference electrode and a platinum wire as a counter electrode. CV measurements were conducted using a potentiostat (263A Princeton Applied Research) and recorded using the PowerCV software (Princeton Applied Research). The cathodal and anodal charge storage capacities (CSC) were calculated using the time integral of the cathodal and anodal current over a potential range defined as the limit for water electrolysis window derived from CV performed at a scan rate of 45 mV/s. The same setup was used to test charge injection for stimulating electrodes, with cathodic-first symmetric biphasic current pulses. The applied pulse width was 50 ms and the inter-pulse delay was 10 ms.

The impedance of the SoftC probe electrode (~50 μm in diameter) was measured at frequencies ranging between 25 and 5,000 Hz in aCSF using RHD2132 recording head stage and an RHD 2000 USB interface board (Intan Technologies LLC, Los Angeles, CA, USA). Electrophysiological data in this study was recorded with a single device in which we monitored stable impedance (274 kΩ at 1 kHz) for 18 recording channels over 120 days. The other 14 channels had higher impedance values (in the range of 10–30 MΩ).

Voltage transients generated by stimulating an electrode of SoftC probe were measured in an aCSF solution. Voltage transient recording was done 50 μm above the electrode surface using a glass capillary electrode filled with 3M KCl, mounted on a computer monitorized micromanipulator (PatchStar, Scientifica) vs. an Ag/AgCl reference electrode in aCSF. The measuring unit consisted of a voltage amplifier (ELC-03XS, npi electronic GmbH). A charge-balanced biphasic current pulse of 400 μs, with increasing amplitudes, was injected to a single stimulating electrode of SoftC probe, using an external stimulator (STG4002, MultiChannel Systems).

### 2.5 Animal care and use

All experimental procedures were performed in accordance with Animal Welfare Law—Experiments in Animals 1994 and under approval by the Institutional Animal Care and Use Committee at Tel Aviv University (permit number: TAU-MD-IL-2207-173-1). Fertilized chick eggs were incubated at 37°C, until embryonic day 14 (E14) followed by rapid egg opening and embryo decapitation. The enucleation and eye preparations were performed in oxygenated (95% O_2_, 5% CO_2_) chick aCSF solution under a binocular microscope. Cornea, lens, sclera, and vitreous humor were removed and for *ex vivo* experiments the retina was detached from the pigmented epithelium, dissected into 4 × 4 mm squares, and laid on the MEAs with the ganglion cell layer (GCL) facing down. To improve coupling between the tissue and the electrodes, a polyester membrane filter (5 μm pores, Sterlitech) and a stainless-steel washer were placed on top of the retina. For the experiments in the eye, the eye cup was carefully transferred to the custom-made holder where the cornea, lens, sclera, vitreous humor, and SoftC probe were gently laid on the surface of the retina. During experiments, the retinas were kept at physiological conditions, at a temperature of 34°C, and perfused (2–5 mL/min) with oxygenated (95% O_2_, 5% CO_2_) chick aCSF solution. In total, five retinas were used for each preparation (*ex-vivo* and intact).

### 2.6 Electrophysiolgy

To reduce the potential for failed experiments, we used two well-characterized devices (softC and TiN MEA) to perform the electrophysiological study. Throughout the entire duration of the study, we monitored the softC device's stability and consistent performance. Impedance measurements were performed before each experiment, following the experiment, the device was thoroughly cleaned with deionized water (DI) and stored. We did not encounter any significant deviations in electrode impedance values or noise levels that could have affected the reliability of our data. Coupling between the tissue and the electrodes was established by gently laying the probe on the surface of the retina, using a manual micromanipulator (Kite-R, World Precision Instruments). The retinas were kept at physiological conditions, at a temperature of 34°C, and perfused (2–5 mL/min) with oxygenated chick aCSF solution. Neuronal signals were amplified and acquired at 25 kHz per channel, with an RHD2132 recording head stage and an RHD2000 USB interface board (Intan Technologies). Retinal stimulation was carried out by injecting a biphasic pulse of 300 μs with an inter-phase delay of 60 μs to a single electrode of the SoftC probe, using electrical stimulus generator (STG4002; MultiChannel Systems). Stimulation amplitudes used in the study varied between 1 and 100 μA at either 1 or 10 Hz stimulation frequency.

Intact retina recordings and stimulation were compared to the *ex-vivo* retina placed on a 30 μm diameter TiN electrode MEAs (MultiChannel Systems)—*ex-vivo* retina model. *Ex-vivo* neuronal signals were amplified with a MEA1060-up amplifier (gain × 1,100, MultiChannel Systems), digitalized using a 64-channel analog to digital converter (MC_Card, MultiChannel Systems), and recorded (MC_Rack, MultiChannel Systems).

The recorded signals were analyzed offline in DataView (software by Heitler W J, version 11.11.1, University of St Andrews, Scotland, UK). Raw traces were first filtered to remove local DC by subtracting a local moving average from the signal, then the signals were filtered with 300–3,000 Hz (Butterworth 2nd order). The stimulation artifact was removed by identifying stimulus onset and interpolating 2.5 or 5 ms before and after the stimulus artifact event for direct and indirect response visualization, respectively. Retinal spikes were detected using threshold-crossing criteria [five times the standard deviation (SD) of noise]. Recorded spikes were processed into individual units using a principal component analysis. Automatic clustering was performed using a built-in unsupervised algorithm by Bouman (1997). Signal-to-noise ratio (SNR) was evaluated for *ex-vivo* and intact retina recordings during 10 Hz stimulation. RMS noise was calculated 80 ms pre-stimulation and RMS signal was calculated in between the stimulation pulses at six random time points (Supplementary Table 1). Intact retina responses to electrical stimulation from all recorded channels is additionally presented in Supplementary Figures 7, 8. Zoom in view to retina responses (both *ex-vivo* and intact) during 10 Hz electrical stimulation is presented in Supplementary Figure 9.

## 3 Results

**Figure 1:**
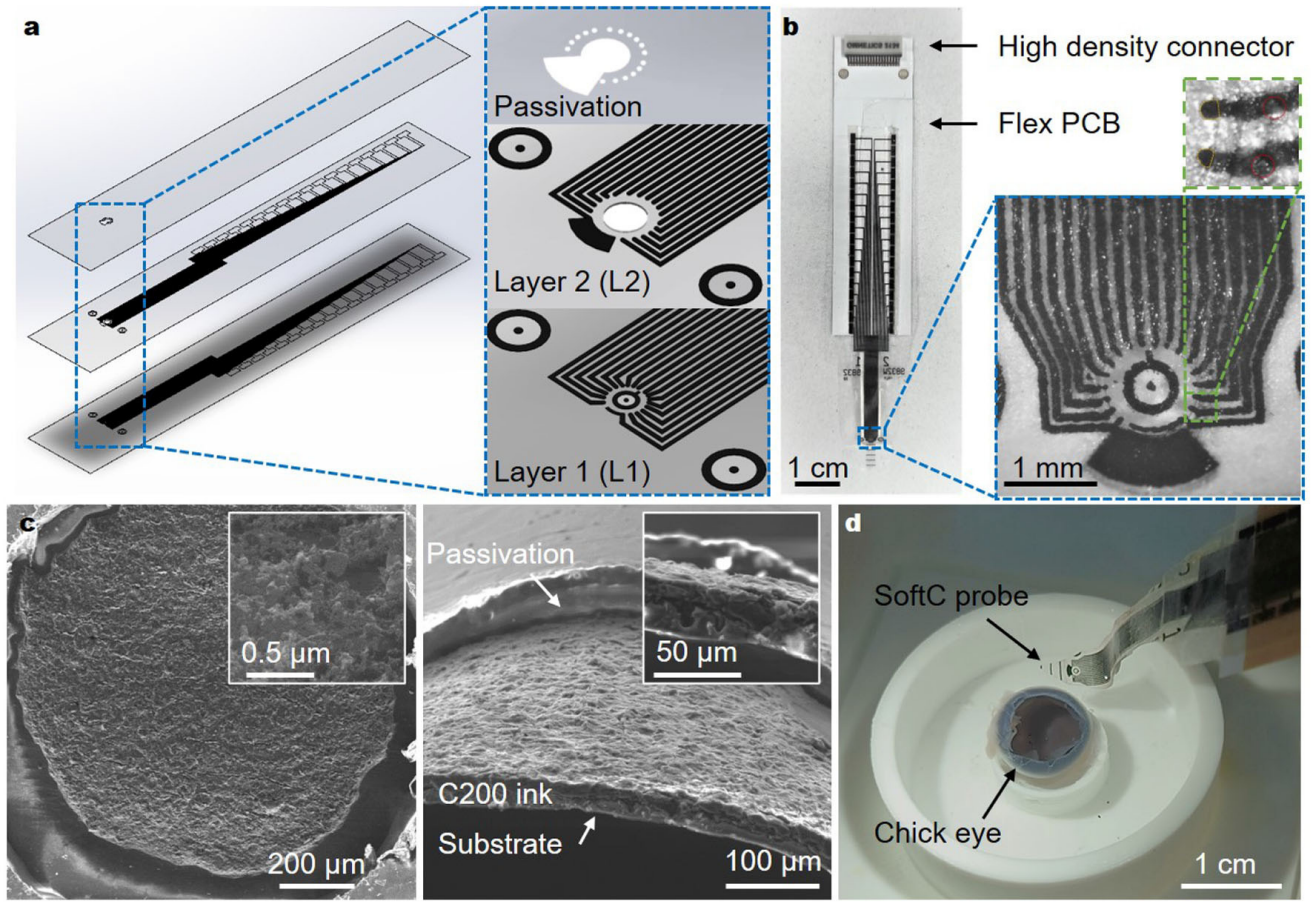
36-Channel SoftC probe. **(a)** The layout of the SoftC probe consists of two layers with printed carbon traces and a top passivation layer. **(b)** Picture of a fabricated complete SoftC probe. Traces are connected to the custom-made flexible PCB and Intan high-density connector. Zoom-in image of the SoftC probe. The top zoom-in image shows four electrodes: two channels (orange circles) of layer 1 and 2 channels (red circles) of layer 2. Red circle diameter 50 μm. **(c)** ESEM images of carbon on PU substrate top (left) and cross-section (right) views. Insets: magnified views. **(d)** Picture showing experimental set-up: chick embryo eye placed in a custom printed holder-chamber, SoftC probe.

Soft electrode arrays (SoftC) were realized using screen printing of carbon ink on soft and thin polyurethane (PU) films. Given the relatively low resolution of screen printing, stacking multiple layers was used to achieve a high electrode count (Figure 1a). Previously, we presented arrays with only eight electrodes (Vėbraitė and Hanein, 2022). In this study, we increased the electrode number to 36 electrodes. We reduced the electrode diameter from 80 to 50 μm and inter-channel distance from 430 to 150 μm, achieving higher spatial resolution and electrode count (Figure 1a). The overall dimensions of the soft array probe are 6 mm × 2.6 cm × 160 μm (width, length, total thickness, respectively) and effective array area of ~ 1.13 mm^2^ incorporating 32 recording-stimulating micro-electrodes, two additional stimulating electrodes, and two big reference electrodes. The final SoftC probe is shown in Figure 1b, consisting of a soft part (with the carbon electrodes), a flexible PCB, and a high-density connector. The electrodes in Layer 2 have a circular shape, as defined by the laser cut hole, and electrodes in Layer 1 have a slightly deformed shape (Figure 1b inset). Electrode's approximate diameter and area were estimated and averaged over five SoftC probes from optical microscopy images (Supplementary Figure 2B), resulting in an overall average diameter of 58.9 μm (SD = 10.7) and an area of 1,475 μm^2^ (SD = 349). Environmental scanning electron microscope (ESEM) imaging was used to examine the electrode surface. Figure 1c shows ESEM images depicting a rough carbon surface, which is important for enhancing an effective electrode surface area. Additionally, a confocal scanning laser microscopy of the carbon on PU film (Supplementary Figure 4) was used to derive a surface roughness of ~1 μm and a height of ~11 μm. Contact angle measurements of the electrode surface (Supplementary Figure 5) demonstrate angles of 32.5 (SD = 2.4) and 81 (SD = 4) for ppEDOT-coated and bare electrodes, respectively. A complete device introduced into an enucleated chick eye is shown in Figure 1d.

**Figure 2:**
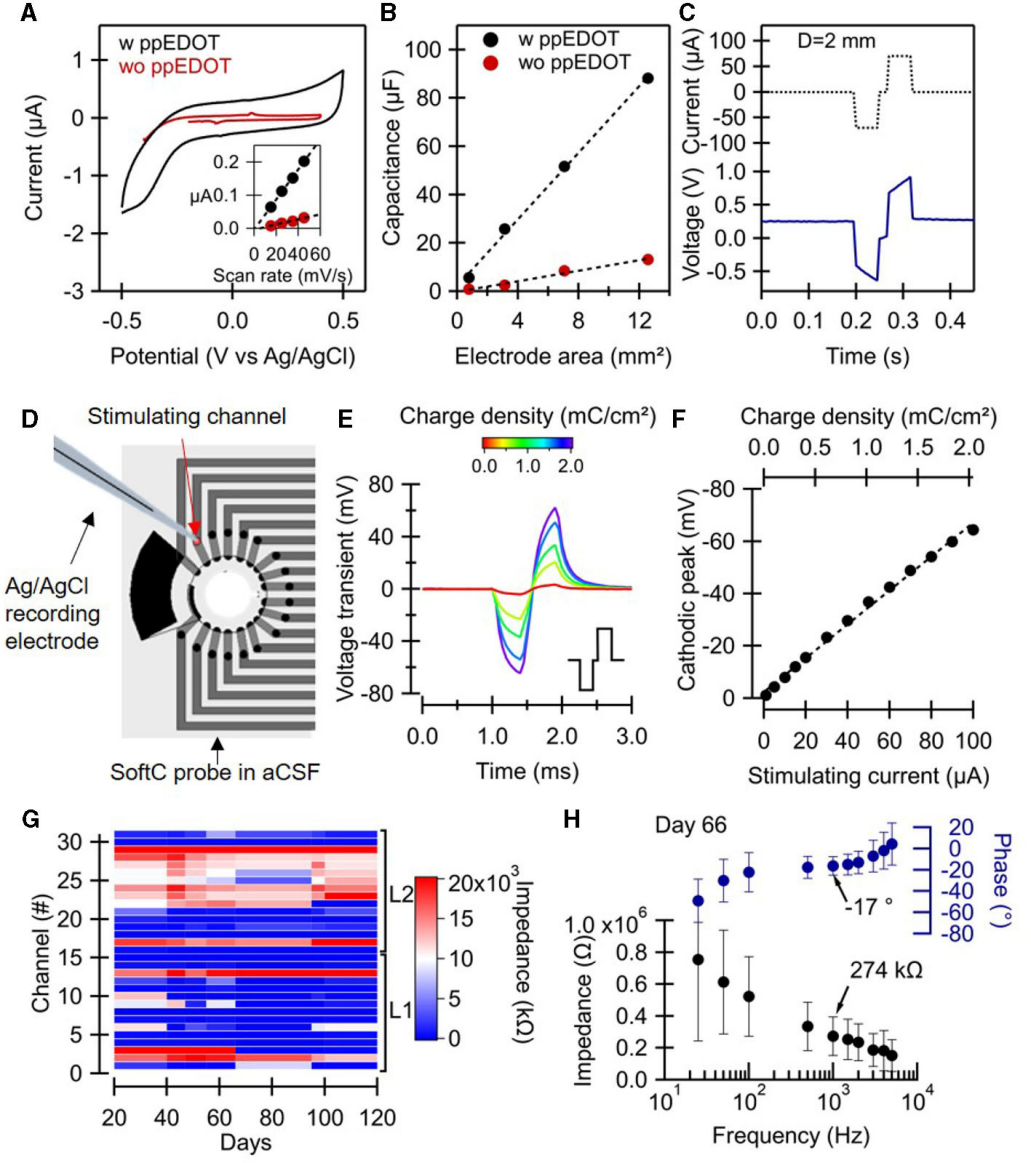
SoftC probe characterization. **(A–C)** Electrochemical characterization was performed on test structures with electrodes 1, 2, 3, and 4 mm in diameter. **(A)** Cyclic voltammetry scan of ppEDOT uncoated and coated electrode (D = 1 mm) under 45 mV/s scan rate. The inset shows the charging current vs. the scan rate of electrodes, dashed lines are linear fits. **(B)** Electrode capacitance vs. electrode area for ppEDOT coated and uncoated electrodes measured under 45 mV/s scan rate. **(C)** Charge injection test with cathodic-first symmetric biphasic 50 ms current pulses. **(D–F)** Voltage transient measurement 50 μm above SoftC probe stimulating electrode with a glass-pulled capillary electrode **(D)** in response to charge-balanced biphasic current pulse (400 μs with 100 μs interpulse delay, e inset) at increasing current amplitudes. **(E)** Transient voltages, Vt, measured in response to biphasic current pulse. **(F)** The cathodic peak of the Vts as a function of the injected current. The equivalent charge density is shown above. **(G, H)** SoftC probe electrode impedance measurements using Intan system in aCSF. **(G)** Impedance magnitude at 1 kHz of all 32 electrodes over time. **(H)** Electrode impedance and phase vs. frequency (25–5,000 Hz). The mean and standard deviation of the impedance of 18 electrodes (channels: 2–11, 13–15, 17, 19–20, 29, 31) were measured on day 66.

Electrochemical measurements were performed to validate electrode performances, including non-Faradaic behavior, specific DC capacitance, impedance, charge injection, and stability (Figure 2). Cyclic voltammetry (CV) of screen-printed electrodes was first used to validate the non-Faradaic nature of the electrodes and to characterize the water window. The low-frequency capacitance of the electrodes was also extracted (Figures 2A, B). In particular, CV measurements reveal the effect of ppEDOT coating. Screen-printed electrodes coated with ppEDOT obtained a specific capacitance of 0.92 mF/cm^2^ (compared with 0.09 mF/cm^2^ for bare electrodes), averaged over five samples each. The high specific capacitance values are important for low-noise electrical recordings at small electrode dimensions. Figure 2A shows that the boundaries of the electrochemical potential window for ppEDOT coated electrode in PBS are −0.5 and +0.5 V (Figure 2A). CV measurements also reveal a capacitive behavior and water window values typical to carbon/ppEDOT electrodes. The cathodal and anodal charge storage capacities (CSC) were calculated by the time integral of the cathodal and anodal current over a potential range determined by a water electrolysis window. Values are presented in the Supplementary Figure 3 for both electrodes. CSC was evaluated to be 2.7 and 2.0 mC/cm^2^ for coated and uncoated carbon electrodes, respectively.

Charge transfer tests were carried out to categorize the stimulating electrodes in terms of their reversibility and maximum charge injection capacity (Figure 2C). The charge injected in the first half-phase is seen to be immediately compensated for by reversing the polarity over the second half. A charge injection test with a specific capacitance of 0.12 mC/cm^2^ (75 μA, 50 ms) shows a potential increase in the 200 mV range at the electrode interface.

To validate the stability of the electrode under electrical stimulation, the potential above the electrode was also measured using a glass-pulled capillary electrode mounted on a motorized stage. Voltage transient *V*_*ts*_ values at different charge densities show an almost linear increase through the 0–2 mC/cm^2^ range. The values of the cathodic peak are similar to those measured at the same distance with commercial 30 μm diameter TiN MEAs (Supplementary Figure 6). In a prior study, screen-printed carbon electrodes on polyurethane films were shown to be compliant with EtOH and ultraviolet (UV) sterilization methods as well as stable in aCSF solution (at 40°C) for over 6 months with no delamination or change in the resistance values (Vėbraitė et al., 2021). Here, the impedance stability of the electrodes in the aCSF solution was validated (Figure 2G) over 120 days. Impedance measurements at the 0.025–5 kHz range on day 66 showed stable values (274 kΩ at 1 kHz).

**Figure 3:**
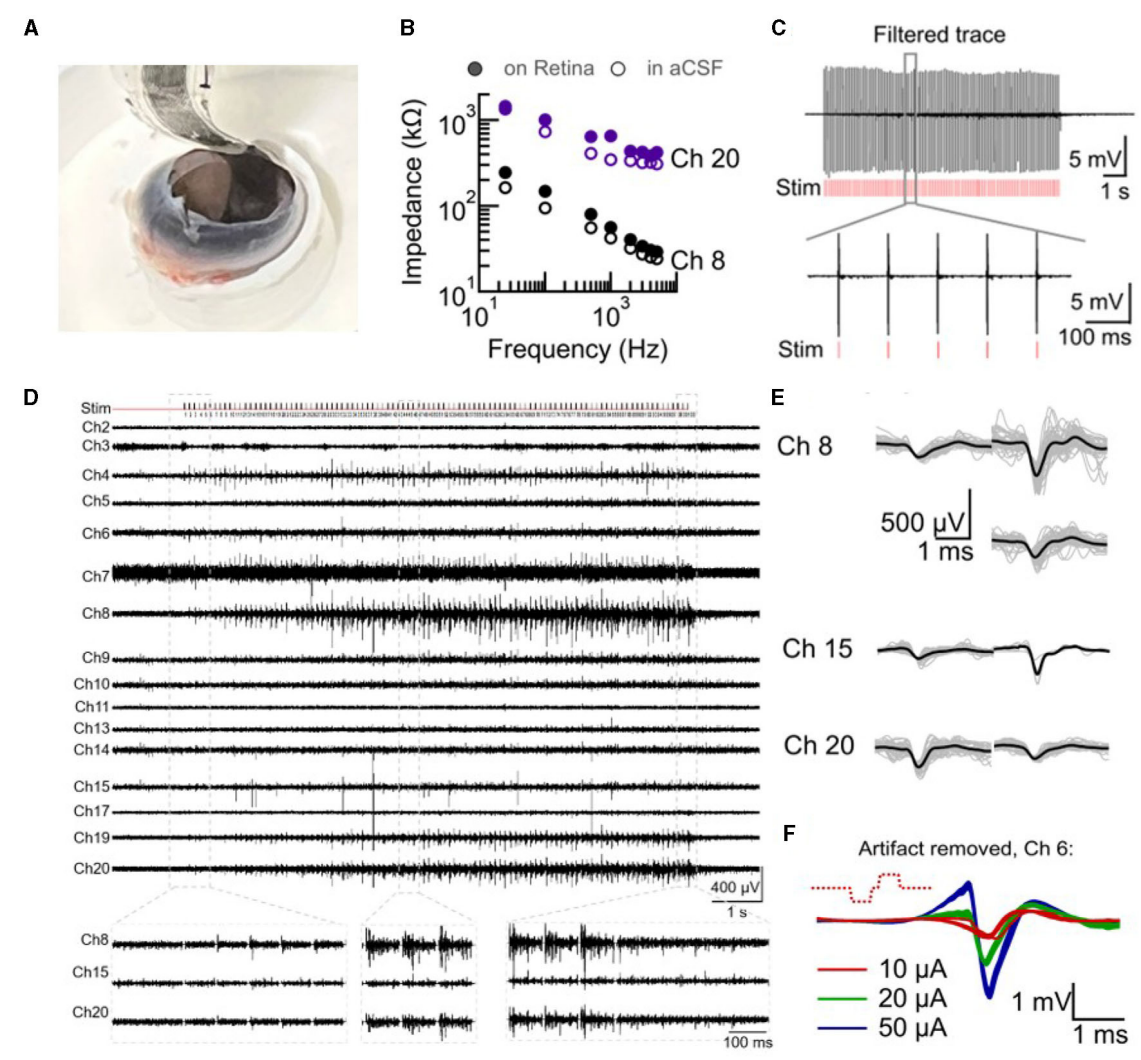
Intact retina recording and stimulation with SoftC probe. **(A)** Picture of 36-channel SoftC probe placed inside the enucleated eye on the intact retina. **(B–E)** Electrical recordings by SoftC probe during a delivery of 50 μA 300 μs charge-balanced biphasic 100 pulse train stimulation at 10 Hz frequency (Retina #1). **(B)** Ch8 and Ch20 impedance amplitude in aCSF before the experiment and after placement on the retina show an increase of 25 and 46% from the initial value at 1 kHz, respectively. **(C)** Second order Butterworth 300–3,000 Hz filtered trace (ch20) before artifact removal. Red vertical lines indicate stimulation onset. Zoomed view to six pulses. **(D)** Electrical recordings of 16 channels over the whole stimulation train after artifact removal (−4 and +4 ms were set to 0 mV at the onset of stimulus). Zoomed view to three different time points of stimulation train of ch8, ch15, and ch20. **(E)** Spiking waveforms detected during the stimulation period. Spike shape classification by principal component analysis (PCA) resulted in 3, 2, and 2 spike waveforms for channels 8, 15, and 20, respectively. Grey lines—10 to 50 spikes superimposed, black line—average waveform. **(F)** Artifact removed (non-filtered trace) short latency responses during 20 μA 300 μs charge-balanced biphasic stimulation at 1 Hz frequency (Retina #2).

Electrical activity in the intact retina in response to electrical stimulation was studied and is presented in Figure 3. A developing chick retina at embryonic day 14 (E14) was used as a blind retina model for these studies (*N* = 5 retinas). Chick retina at E10–E18 has developed and functional ganglion cell layer as well as inner and outer layers but an undeveloped photoreceptor layer with no responses to light until E19 (Mey and Thanos, 2000; Sernagor et al., 2001). The soft probe was placed inside an open blind chick eye (E14) mounted on a custom-made holder (Figure 3A). The eye was kept submerged in oxygenated aCSF (for as long as 3 h).

Electrode impedance measurements were first performed in aCSF and after placement against the retina to validate tissue-electrode coupling (Spira and Hai, 2013; Majdi et al., 2014). The measured impedance increased or remained the same. In six channels (out of 16 measured) impedance values (at 1 kHz) increased by 4–50% from the baseline value measured in aCSF (Figure 3B), indicating coupling. Bi-directional measurements were then used to study retina responses to electrical stimulation. For all SoftC probe stimulations of the intact retina, 300 μs charge balance biphasic pulses were used at either 1 or 10 Hz frequency. Figure 3C illustrates recorded traces contaminated with stimulation artifact, thus it was removed during offline analysis. The filtered and artifact-removed (see Section 2) data reveal a clear retina response to electrical stimulation. Both direct (Figure 3F) and indirect activation (Figures 3C–E) were observed. The first is typified by short, high-amplitude responses that increase with increasing current amplitude. Indirect activation is characterized by low-amplitude and long latency responses. We note that retina responses were clearly visible in those channels with improved coupling (e.g., channels 15, 20, and 8 with 6, 25, and 46% increase in impedance, respectively). These results suggest that despite the curved nature of the intact retina, the soft probe achieved good electrical coupling, especially in those electrodes with high signal-to-noise ratio responses.

**Figure 4:**
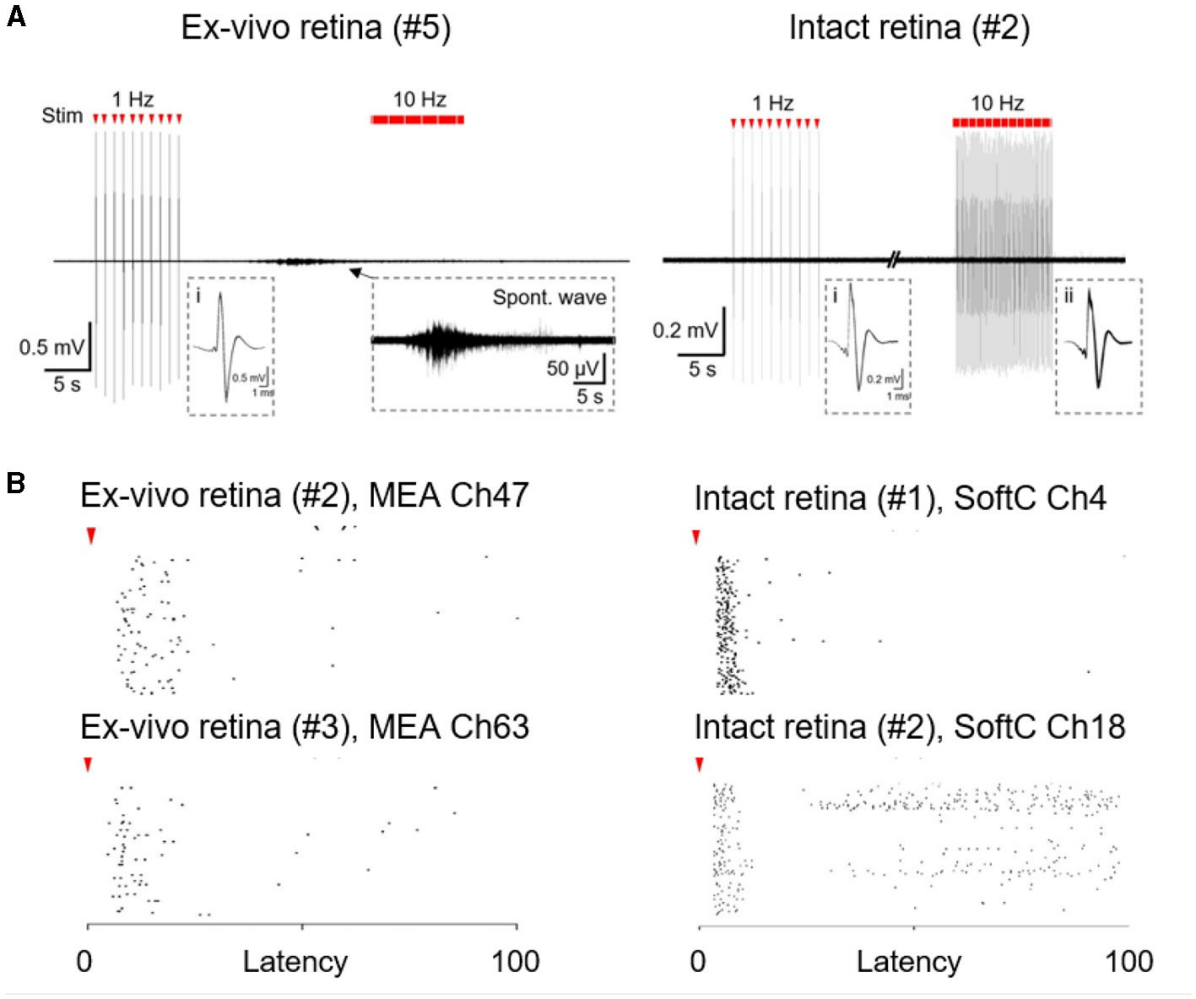
Spontaneous and stimulated retina activity. **(A)** Responses to electrical stimulation in *ex-vivo* and intact retina to 1.69 and 0.76 mC/cm^2^ stimulation, respectively. A typical spontaneous wave in *ex-vivo* retina recordings (left, zoom in inset). No such activity was observed in intact retinas (right). Insets (i-ii) superimposed direct responses. **(B)** Stimulus-response (raster plots) to 10 Hz stimulation over 10 s in the *ex-vivo* retina (left; 0.68 and 1.49 mC/cm^2^ stimulation for top and bottom, respectively ) and in the intact retina (right; 0.76 mC/cm^2^ stimulation). Each row corresponds to 100 consecutive stimulation pulses. Red arrowheads indicate stimulus onset.

Finally, we compare the indirect responses (long latency) in the intact retina with *ex-vivo* conditions. In the intact retina, responses appear to remain stable upon repetitive stimulation lasting for 10 s (Figures 3D, 4A, B). Similar stimulation in *ex-vivo* conditions is typified by clear desensitization, especially with higher stimulation frequencies (Jensen and Rizzo, 2007; Chenais et al., 2021; Li et al., 2022), and a strong sensitivity to spontaneous activity, which completely abolish the response to stimulation. Figure 4A presents an example of a spontaneous wave direct inhibition of retina responses to electrical stimulation in the *ex-vivo* retina, whereas in the intact retina, such an effect was not detected. We observed almost no spontaneous activity [Figure 4A (right)] in the intact retina experiments (*N* = 5 retinas). This is, to the best of our knowledge, the first direct electrophysiological evidence for a discrepancy between *ex-vivo* and intact retina conditions.

## 4 Discussion

Soft electrode arrays, engineered for bi-directional electrical interfacing with the retina, were realized and put through testing. The probes were realized by stacking two screen-printed soft layers. We were able to increase the electrode count and realize electrode arrays with good recording and stimulation performances, even when positioned against the intact retina. Various flexible and soft probes were investigated in the past. We briefly discuss a few representative examples. In Graudejus et al. (2009), 11 recording electrodes were realized using photolithography and electrodeposition on 280 μm thick layer of an elastomeric silicone substrate. Silicon nanomembrane transistor arrays on polyimide (few μm thick, Young's modulus 1.3–3 GPa) were used to form dense arrays of thousands of amplified and multiplexed sensors (Viventi et al., 2011). The use of a grid structure was also used to help reduce the rigidity of a recording device as demonstrated in Reference (Khodagholy et al., 2015). These and many other approaches have not demonstrated high-density recording and stimulation with thin and soft support. Transitioning from rigid or flexible substrates to soft ones usually comes with a cost in performance. For example, CNT micro-electrodes (30 μm in diameter) on Si/SiO_2_, can reach high specific capacitance in the range of 3–10 mF/cm^2^ (Gabay et al., 2007). Values obtained with state-of-the-art Titanium Nitride (TiN) on glass are in the 2 *mF*/*cm*^2^ (Gabay et al., 2007). CNT micro-electrodes on soft support will typically have a specific capacitance in the range of 1–2 mF/cm^2^. ECoG probe using CNTs on PDMS electrodes exhibits specific capacitance of 1.5 mF/cm^2^, low impedance values of approximately 30 kΩ at 1 kHz, and charge storage capacity of 0.35 mC/cm^2^ (Yang et al., 2022). Faradaic silver nanoparticles/PEDOT:PSS [Poly(3,4-ethylenedioxythiophene) Polystyrene Sulfonate] electrodes on polyimide can reach a very low impedance value of 200 Ω (at 1 kHz), exhibit capacitance of 0.4–60.6 mF/cm^2^ and charge storage capacity of 1.083–2.8 C/cm^2^ by increasing PEDOT:PSS coating from 1 to 10 layers (Almaris et al., 2020). The soft and thin electrode arrays we presented here achieve good recording and stimulation performances despite their very simple and straightforward fabrication process and as such represent an advantage compared with alternative approaches.

These pliable probes are particularly adapted to conform to the curvature of the eye's retina, providing a distinctive chance to investigate the retina in its undisturbed state. Using the SoftC technology, we demonstrated, for the first time, electrophysiological differences associated with retina conditions (i.e., *ex-vivo* vs. intact retina). Differences associated with spontaneous activity were observed thanks to the ability to record directly from the intact retina. Differences associated with evoked responses were facilitated by the bi-directional ability presented here.

Discrepancies between *in vitro, ex-vivo*, and intact tissues are well documented (Alaylioğlu et al., 2020). *Ex-vivo* retinas, in particular, are expected to show some structural damage and decreased functional responses compared to intact retinas, owing to mechanical manipulation and the loss of optimal biochemical support. These differences imply that the *ex-vivo* preparation may not accurately reflect the *in vivo* state of the retina. Among various expected effects, these differences are expected to profoundly affect retina electrophysiology.

One such effect is spontaneous waves. Retina spontaneous activity is a well-established phenomenon of developing and degenerating retinas and is known to interfere with retina stimulation strategies (Wong et al., 1998; Goo et al., 2016; Haselier et al., 2017). In a previous study (Vėbraitė et al., 2021), we presented bio-potential measurements from intact retinas. In those measurements, spontaneous activity was observed in only 2 out of 11 intact eyes. On those rare appearances, the recorded spontaneous activity was shorter in duration and had a low signal-to-noise ratio compared to that typically observed *ex-vivo*. In our previous study, the SoftC probes did not have electrical stimulation capability, and the observation of spontaneous retinal activity was an indicator of good probe-retina interface and retina viability. In this study, we report almost no spontaneous activity in the intact retina (*N* = 5 retinas). Importantly, in the present study, the ability to electrically stimulate retina responses serves as a powerful tool to validate retina viability and to provide greater confidence in asserting the lack of spontaneous activity in intact retina.

Studying retina responses to electrical stimulation is pivotal in the domain of artificial vision (also known as retinal prosthesis or a bionic eye). Electrical stimulation works by using electrodes implanted in the retina to stimulate remaining healthy cells and transmit visual information to the brain (Bloch et al., 2019; Ayton et al., 2020). For individuals with severe vision loss, electrical stimulation of the retina can provide a means of regaining functional vision, allowing them to perform basic daily activities, such as recognizing faces, reading, and navigating the environment (Castaldi et al., 2016; Hallum and Dakin, 2021). Electrical stimulation of the retina has shown promising potential in treating certain forms of visual impairments, particularly retinal degenerative diseases such as retinitis pigmentosa and age-related macular degeneration (Chow et al., 2002; Palanker et al., 2022). There are several lingering challenges related to electrical stimulation of the retina, primarily: Stability, efficacy, safety, and feedback (Ryu et al., 2019). Having demonstrated the intact retina responses to electrical stimulation, it is interesting to discuss these results focusing on stability, efficacy, and safety in comparison to the extensively published *ex-vivo* retina data.

In this investigation, we were able to observe both direct and indirect responses in the intact retina. Interestingly, the responses observed in the intact retina remain stable, even after extensive stimulation (Figures 3D, 4B). Similar stimulation in *ex-vivo* retina is typified by clear saturation (Chenais et al., 2021). We hypothesize that the origin of this interesting discrepancy is the physiological state of the retina, which gives rise to better stability of the intact retina under what appears to be better conditions. In the realm of retina stimulation, high efficacy stands for the ability to achieve local stimulation with high temporal resolution. We expect that ganglion and bipolar cell response to stimulation, and in particular, its efficacy will be affected by whether retina circuits are intact or have lost their natural support. These issues will be further discussed and analyzed in a separate publication.

A major concern in electrical neural stimulation is safety. Moreover, the current or charge delivered has to be minimal. Identifying optimal parameters for high-fidelity cell activation is still an active research question (Colodetti et al., 2007; Madugula et al., 2022). Retina activation (direct or indirect) effectiveness is influenced by stimulation parameters such as waveform shape, amplitude, duration, frequency, and polarity (Tong et al., 2020). For example, some studies point out the advantages of monophasic stimulation while others suggest that biphasic stimulation shows a lower activation threshold and shorter response latency. Generally, biphasic stimulation is widely used for retinal implants (Jensen and Rizzo, 2009; Boinagrov et al., 2014; Jalligampala et al., 2017; Celik and Karagoz, 2018; Meng et al., 2018). Another safety concern is oxidative stress. Overproduction of reactive oxygen species (ROS) is known to affect the electrode-tissue interface, which can lead to foreign body response, damage to the cells and tissue, and can also create structural damage, and changes in properties of the implanted device (Takmakov et al., 2015; Ereifej et al., 2018). The retina is particularly sensitive to ROS, which can lead to a range of retinal disorders *in vivo*, including age-related macular degeneration (AMD), diabetic retinopathy, and retinitis pigmentosa, among others (Wang et al., 2022). Thus, the retina exhibits several protective mechanisms, including the presence of antioxidants such as glutathione, vitamins, and taurine as well as enzymes such as superoxide dismutase and catalase. Additionally, the retina's high metabolic rate and high oxygen consumption are regulated to minimize the production of ROS (Léveillard and Sahel, 2017; Domènech and Marfany, 2020). It is therefore not too surprising that extensive stimulation *ex-vivo* could lead to various instabilities, some of which may be associated with the electro-chemistry at the electrode interface unless electrode passivation is explicitly used (Fromherz and Stett, 1995; Eickenscheidt et al., 2012). How more efficient stability is offered by *in-vivo* conditions is an important topic for further investigations.

An additional clear bottleneck in the field of retina stimulation is the lack of informative feedback on the efficacy of a specific stimulation paradigm. Optimized stimulation parameters (e.g., pulse duration, shape, and rate) are important to reduce threshold and energy, provide more selective stimulation (ganglion vs. bipolar cells), reduce fading, and increase localization. In pre-clinical stages, researchers have to rely on animal behavior, evoked potential responses, or data collected from dissociated retinas. These parameters provide only indirect information. For example, the selectivity between ganglion cell, fiber layer, and bipolar cell stimulation has received much attention (Tong et al., 2020), as it is hypothesized that axon fiber activation contributes to reduced localization. Moreover, reports on optimal stimulation parameters are contradictory. Some studies suggested that particularly long (10 ms) pulses can lead to selective activation of bipolar cells (Boinagrov et al., 2014). While Tong et al. (2019), showed that long pulses do not lead to the desired selectivity. One possible explanation for the discrepancy between reports is electrode geometry. An alternative explanation is the different physiological conditions of the retina. Exploring this effect in the intact retina will help in understanding the role of retina physiology in retina stimulation. Desensitization, owing to rapid stimulation, is another major unresolved issue in neural stimulation. In particular, the effect of pulse trains on ganglion cells vs. bipolar cells. The technique reported here can help alleviate the uncertainty associated with electrical stimulation of the retina and will be used in future explorations.

The probes presented in this study can benefit a wide range of neurotechnological applications, far and beyond the study of the retina. Despite the fabrication simplicity, the arrays are stable while also manifesting high-quality performances for bi-directional electrical interfacing. This can be handy in the realm of cortical electrodes, subdermal implants, and much more.

Despite its numerous advantages, the technology presented in its current form is not without drawbacks. One notable limitation is the yield of the manual printing and layer stacking procedures.

An additional challenge is the probe thickness and lack of permeability. Enhancements in probe design, including reducing the thickness of the device, incorporating perforations to increase oxygenation, and maintaining uniformity throughout the fabrication process, coupled with a more extensive validation process both in laboratory settings and within living organisms, will provide stronger support for establishing the showcased technology as a resilient instrument in upcoming research endeavors.

To summarize, this feasibility study demonstrates a novel approach featuring a high-resolution probe for electrophysiological investigation of the intact retina. We conducted electrical stimulation while simultaneously recording both direct and indirect responses with the same device. Our findings revealed low levels of spontaneous activity and stable responses under high-frequency stimulation compared to *ex-vivo* retinas. These initial observations suggest distinctions between *ex-vivo* and intact retina conditions. However, further investigations are warranted to comprehensively explore the underlying mechanisms responsible for these differences.

## Data availability statement

The raw data supporting the conclusions of this article will be made available by the authors, without undue reservation.

## Ethics statement

The animal study was approved by Institutional Animal Care and Use Committee at Tel Aviv University. The study was conducted in accordance with the local legislation and institutional requirements.

## Author contributions

IV: Conceptualization, Investigation, Writing—original draft, Writing—review & editing. CB-H: Investigation, Writing—review & editing. MD-P: Investigation, Conceptualization, Writing—review & editing. YH: Conceptualization, Funding acquisition, Supervision, Writing—original draft, Writing—review & editing.
