# Supplementary material for: Bi-directional electrical recording and stimulation of the intact retina with a screen-printed soft probe: a feasibility study
Source: Front Neurosci. 2024 Jan 8;17:1288069. doi: 10.3389/fnins.2023.1288069 (PMC10804455; doi:10.3389/fnins.2023.1288069)
Supplement: Supplementary file 1 [file Data_Sheet_1.PDF]

Supplementary Material

1 SUPPLEMENTARY FIGURES

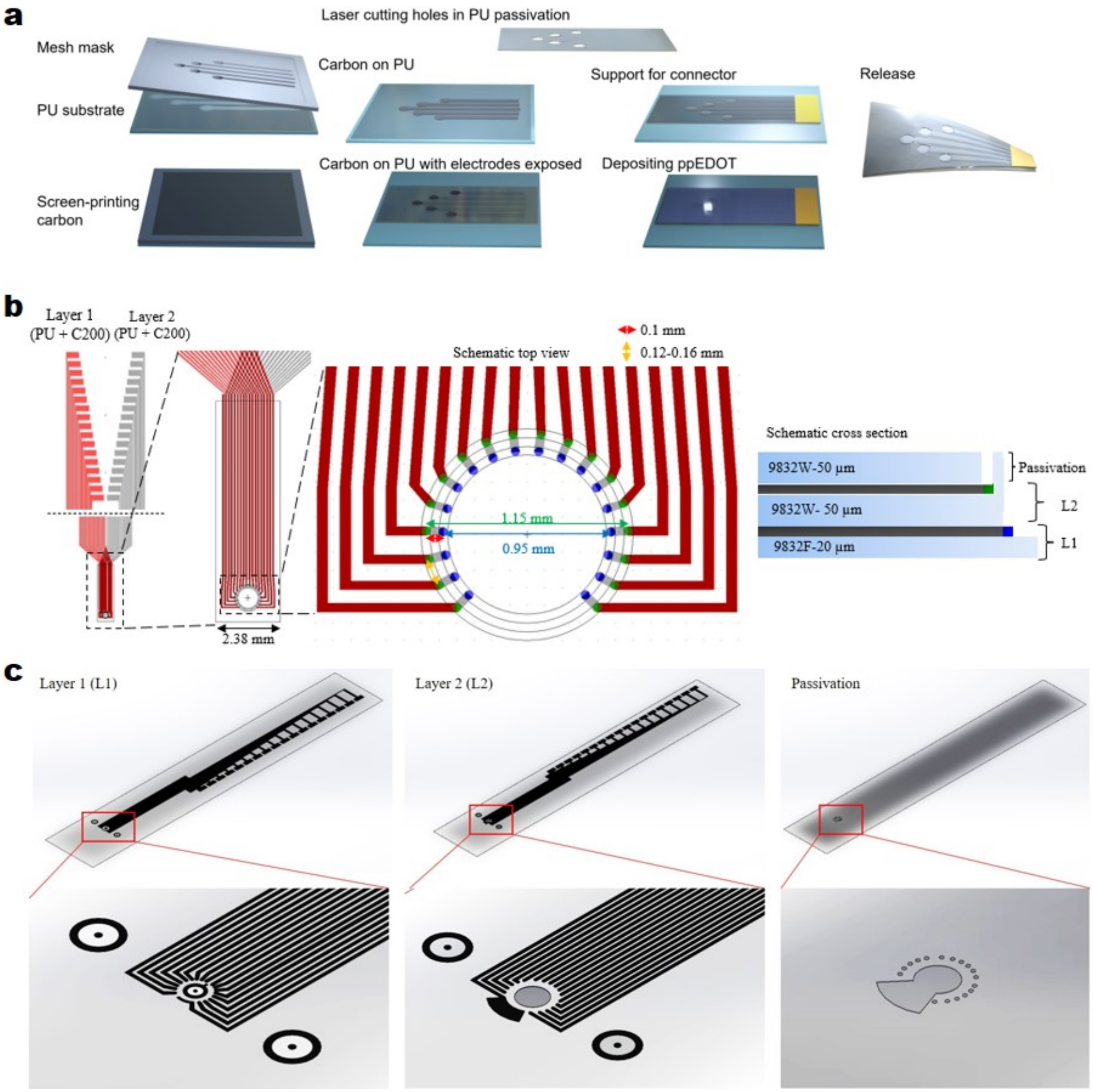

Figure S1. Schematics and graphics of SoftC 36-Channel Probe and PCB Design. (a) Simplified graphics of SoftC electrode array fabrication. Process includes: PU film alignment with the screen print mask, carbon ink application onto the mask surface and screen printing using a 70 durometer squeegee, laser cutting the passivation layer, alignment, and attachment to the printed PU film, addition of support, ppEDOT deposition, and release. In softC 36ch probe, an additional step is included to produce a second layer consisting of the electrode traces and a circular hole in the center to expose electrodes on the first layer. (b) Schematics of SoftC 36 ch. probe with approximate dimensions. Design of custom-made flexible PCB board with Omnetics connector to connect to the RHD2132 recording head stage. (c) Graphics of SoftC 36ch probe layers. Bottom row - zoom in.

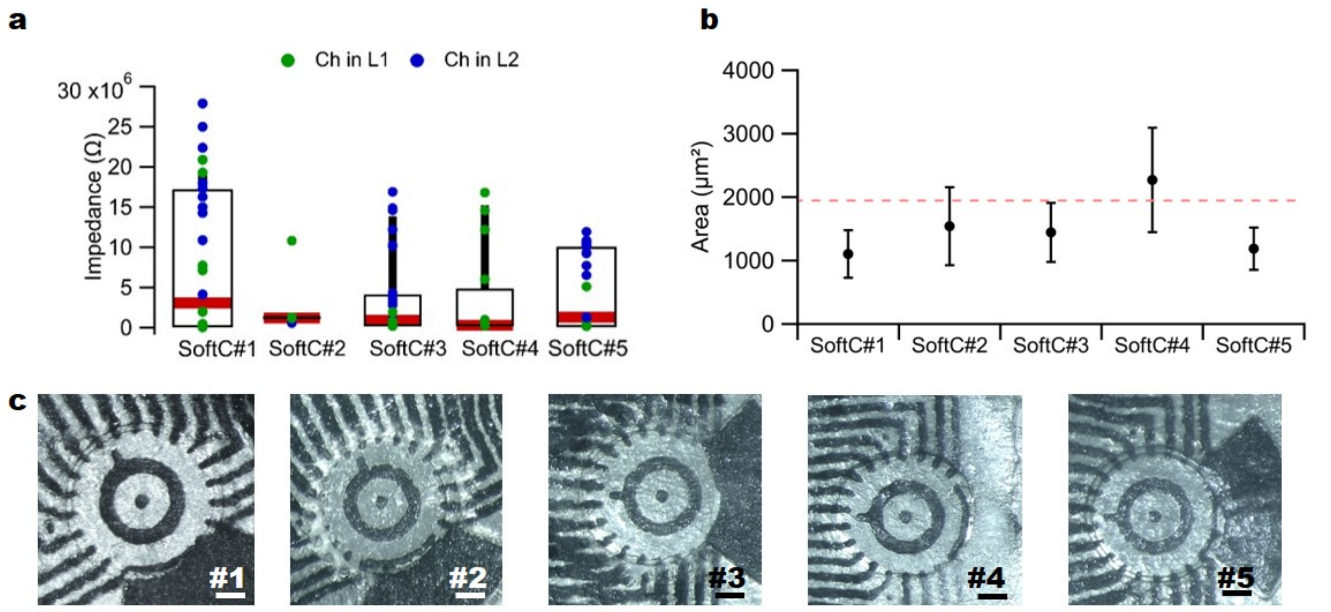

Figure S2. Impedance Characteristics and Channel Area Analysis in SoftC Probes. (a) A box and whisker plot of impedance values of 5 SoftC probes. Green markers - channels in layer 1 (L1), blue markers - channels in layer 2 (L2). SoftC#4 had only layer 1 attached, consisting of 16 recording/stimulating channels, 1 additional stimulating channel, and 1 ground. The red mid-line shows the median value for each SoftC probe, the top and bottom show the upper and lower quartiles (the 75th and 25th percentiles) and the whiskers show extreme values. (b) Channel average area calculated from the pictures in (c) in each SoftC probe. Channels that were covered by passivating layers due to misalignment were not considered in the calculation, number of channels taken into the calculation: SoftC#1 - 34, SoftC#2 - 30, SoftC#3 - 29, SoftC#4 - 15, SoftC#5 - 34. The Red dashed line indicates the ideal channel area when the diameter is 50 μm.(c) Images of SoftC#1 - #5 probes. Scale bar - 200 μm.

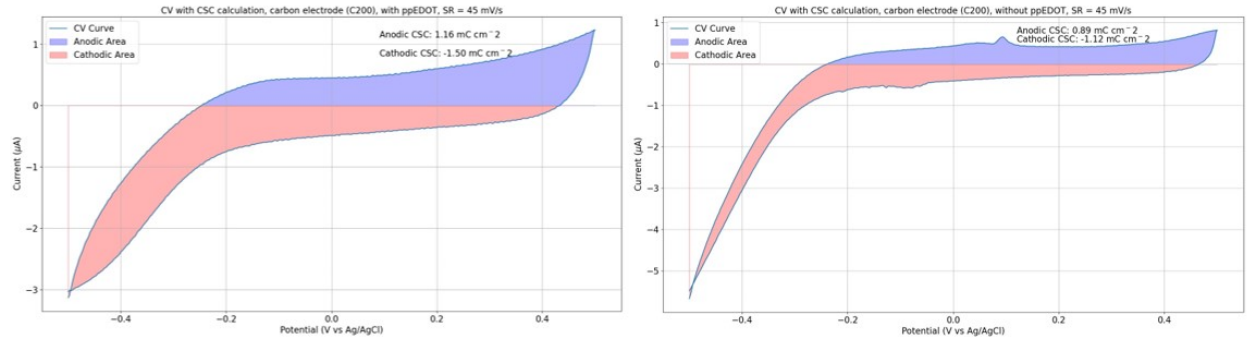

Figure S3. Assessment of charge storage capacities (CSC) for screen printed carbon electrodes with (left) and without ppEDOT (right). CVs have been performed in PBS within -0.5 and $+0.5$ V sweep window with a constant scan rate of 45 mV/s. The cathodal and anodal CSC (mC/cm^2) were calculated by the time integral of the cathodal and anodal current over a potential range of water electrolysis window. Values are presented in the figures for both electrodes. CSC total for ppEDOT-coated carbon electrodes was evaluated to be 2.66 mC/cm^2 and 2.01 mC/cm^2 for uncoated electrodes.

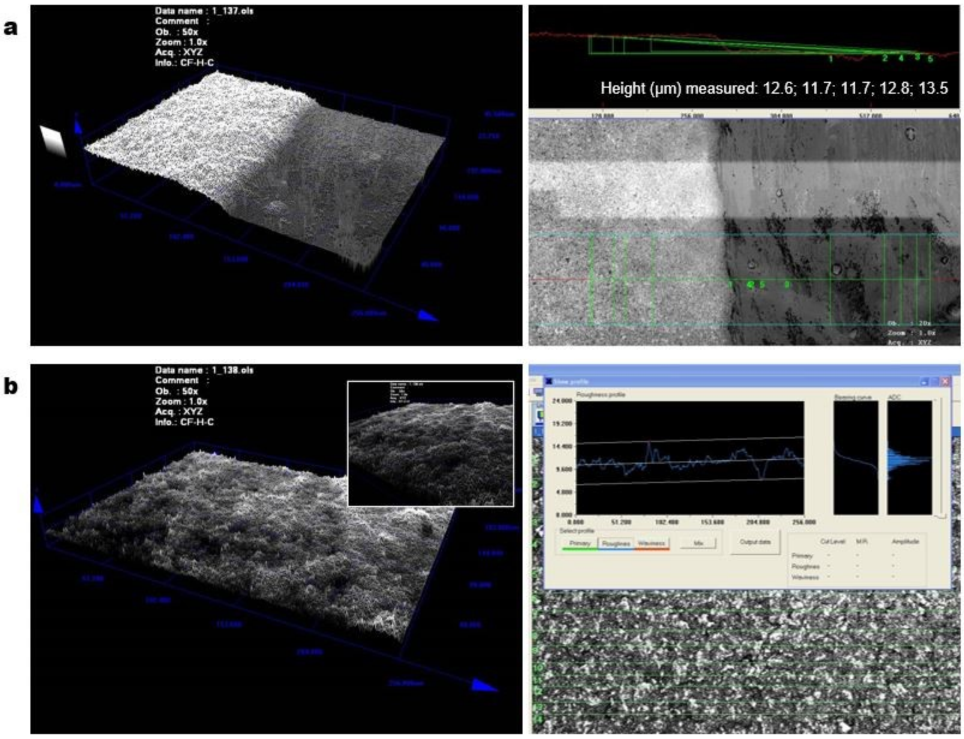

Figure S4. Confocal laser microscopy images of carbon on PU film showing (a) carbon height and (b) roughness.

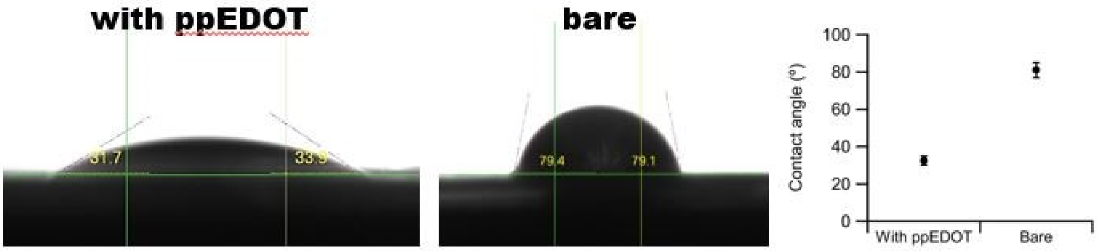

Figure S5. Water contact angle analysis of screen-printed carbon electrodes. Water contact angle images and averaged values (n = 6 measurements) of bare and ppEDOT-coated screen-printed carbon electrodes.

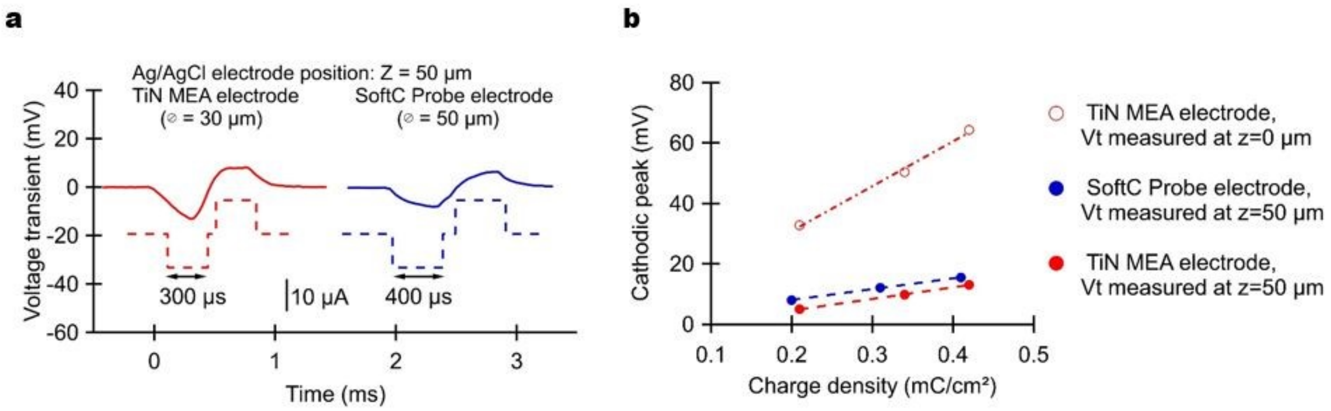

Figure S6. Voltage Transients and Cathodic Peaks Comparison between TiN MEA and SoftC probe electrodes. (a) Transient voltages, Vts, measured with a glass capillary electrode placed 50 μm above a 30 μm diameter TiN MEA electrode and 50 μm diameter SoftC probe electrode. Biphasic current pulse of 10 μA, 300 μs (left inset, for TiN MEA) and 10 μA, 400 μs (right inset, for SoftC probe) were injected into a single electrode. (b) The cathodic peak of the transient voltages as a function of the charge density, measured 50 μm above the TiN MEA and SoftC probe electrode. Additionally, cathodic peak values measured 0 μm above the TiN MEA added.

Table S1. Examples of signal-to-noise ratio (SNR) in five intact and five ex-vivo retina experiments. A single channel was analyzed for each (channel number is indicated in the first column). SNR and Signal values were calculated from 6 random time points (80 ms windows, away from the stimulation artifact) during 10 Hz electrical stimulation (in between stimulation). Noise values were obtained from the 80 ms time point before stimulation onset.

Retina	Noise RMS	Signal RMS $\pm SD$	SNR	Presented in Figure
Intact Retina #1 (Ch20)	16.91	36.82 ± 6.76	2.18	Fig. 3c-e, Fig 4b (top)
Intact Retina #2 (Ch18)	10.15	45.61 ± 28.59	4.49	Fig. 3f, Fig. 4a, Fig. 4b (bottom)
Intact Retina #3 (Ch20)	12.67	51.22 ± 15.33	4.04	Fig. S8, S9
Intact Retina #4 (Ch30)	7.84	12.87 ± 1.83	1.64	Fig. S8, S9
Intact Retina #5 (Ch19)	4.09	7.17 ± 1.51	1.75	Fig. S8
Ex-vivo Retina #1 (Ch28)	2.56	3.49 ± 0.42	1.36	Fig. 4a
Ex-vivo Retina #2 (Ch47)	2.37	3.64 ± 0.53	1.54	Fig. 4b (top)
Ex-vivo Retina #3 (Ch63)	2.52	3.64 ± 0.23	1.44	Fig. 4b (bottom), Fig. S9
Ex-vivo Retina #4 (Ch36)	2.37	3.24 ± 0.2	1.36	Fig. S9
Ex-vivo Retina #5 (Ch83)	2.38	4.84 ± 1.24	2.02	Fig. S9

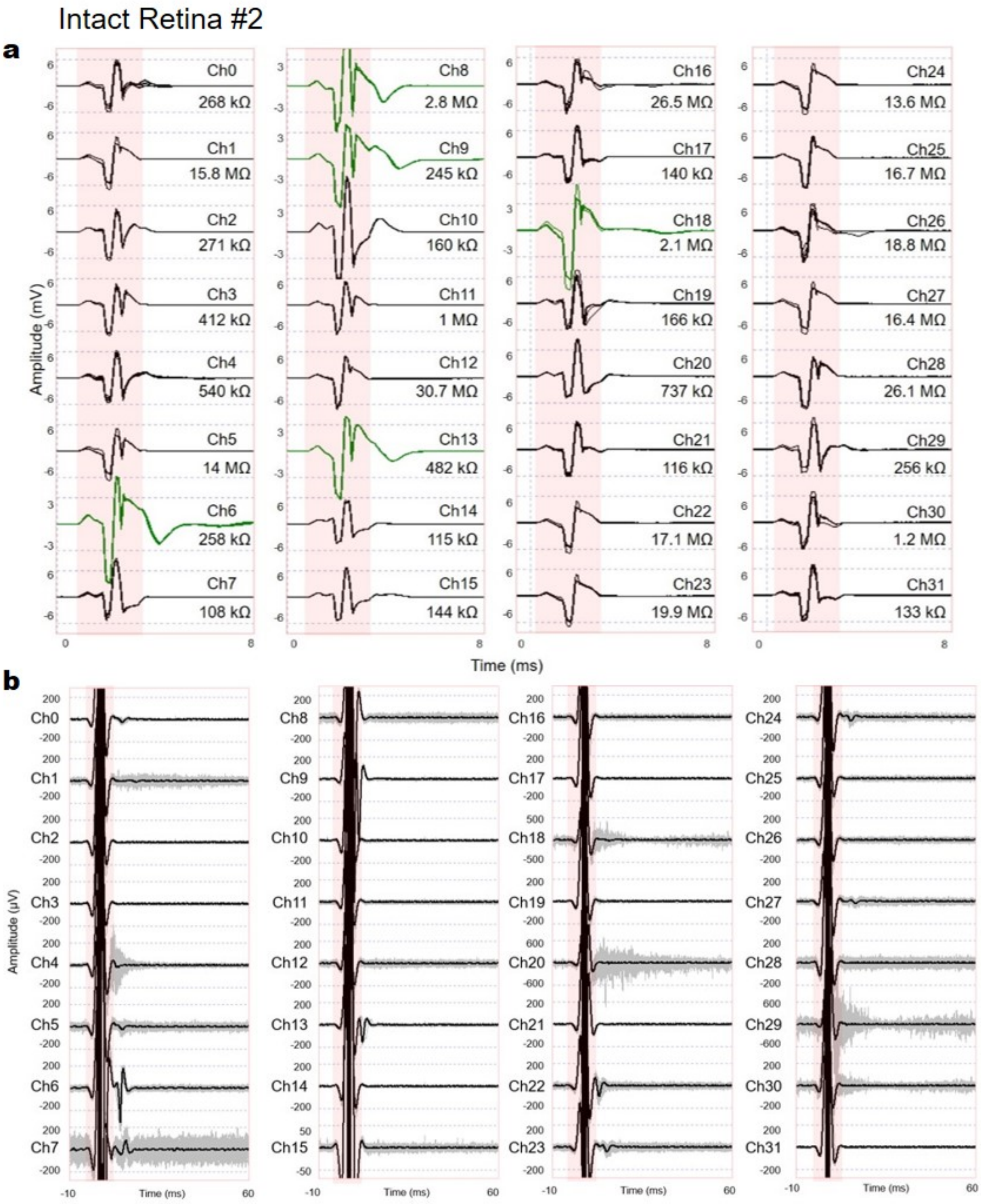

Figure S7. Intact Retina #2 responses to electrical stimulation. (a) Data from all 32 channels during 20 μA 300 μs charge-balanced biphasic stimulation at 1 Hz frequency. Each channel displays 10 superimposed pulses. The red shadow indicates stimulation artifact area. Direct responses are marked in Green traces (ch6, 8, 9, 13, 18). Each channel impedance measured at 1 kHz is displayed. Channel 6 data with 10, 20, and 50 μA is presented in Figure 3f. (b) Data from all 32 channels during 50 μA 300 μs charge-balanced biphasic stimulation at 10 Hz frequency. Raw traces were filtered with 2nd order Butterworth filter (300-3000 Hz). Each channel displays 100 superimposed pulses grey lines). Red shadow indicates the artifact area. Black line – average signal over 100 pulses.

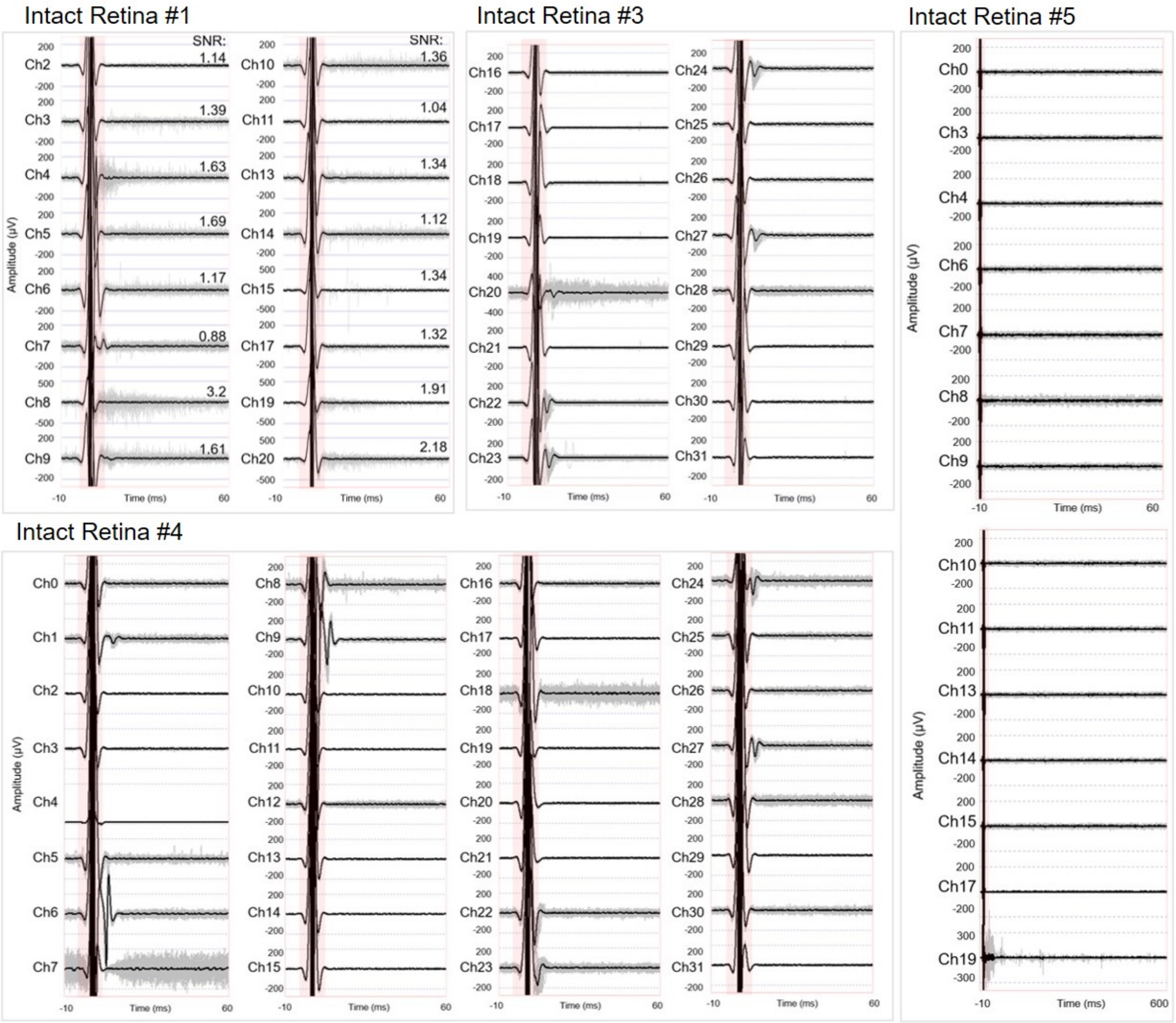

Figure S8. Intact Retina #1, #3, #4 and #5 responses to electrical stimulation. Data presented here is from all channels that were used to record. Raw traces were filtered with 2nd order Butterworth filter (300-3000 Hz). Intact Retina #1, #3, #4: 50 μA 300 μs charge-balanced biphasic stimulation at 10 Hz frequency, each channel displays 100 superimposed pulses (grey) and averaged signal (black line). Intact Retina #5: 100 μA 300 μs charge-balanced biphasic stimulation at 1 Hz frequency, each channel displays 10 superimposed pulses (grey) and averaged signal (black line).

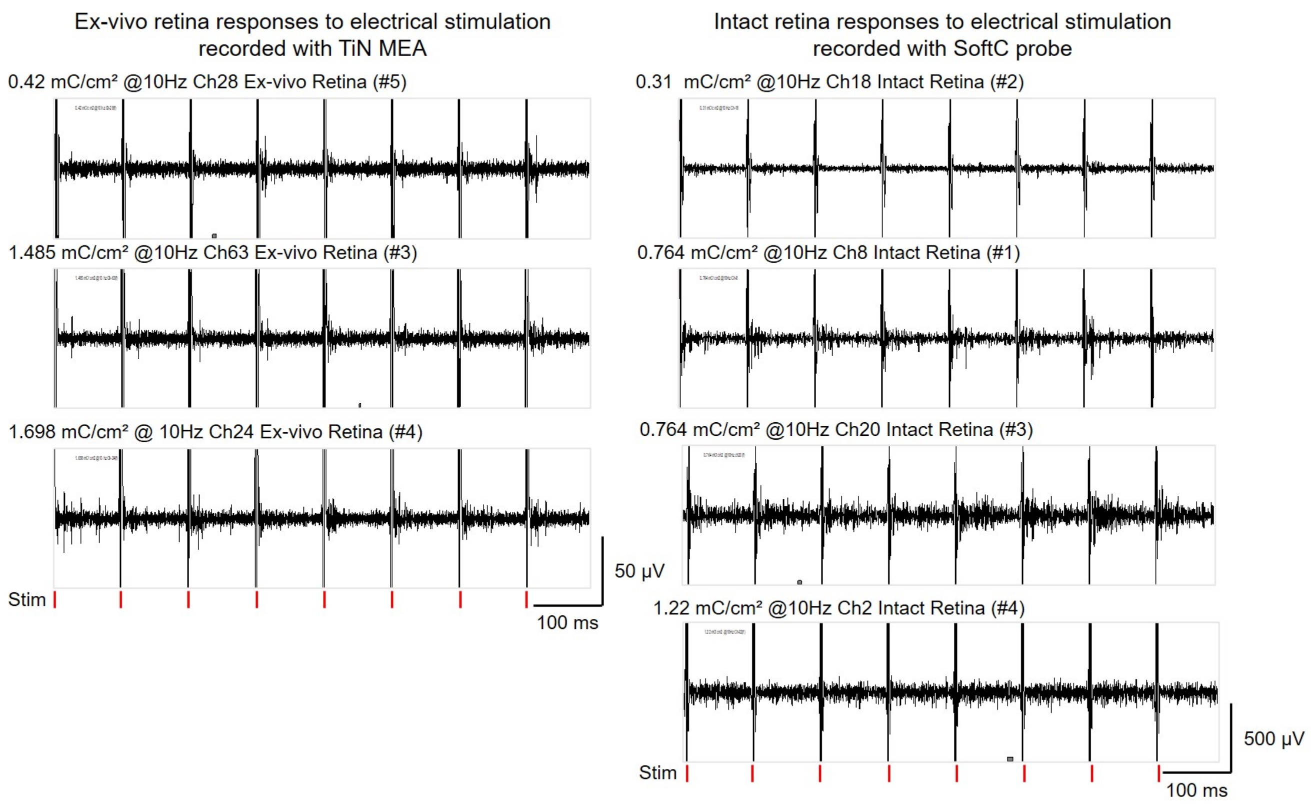

Figure S9. Long latency responses comparison between ex-vivo and intact Retina during electrical stimulation. Data presented here is from a single experiment single channel at a random timepoint during 10 Hz 100 pulse stimulation. Raw traces were filtered with 2nd order Butterworth filter (300-3000 Hz). Red vertical lines indicate stimulation onset.
